# Defining the profile of ***International Archives of Medicine***

**DOI:** 10.1186/1755-7682-1-1

**Published:** 2008-04-15

**Authors:** Manuel Menendez-Gonzalez

**Affiliations:** 1 Department of Internal Medicine, Unit of Neurology, Hospital Alvarez-Buylla, Mieres, Asturias, Spain

## 

This editorial accompanies the launch of *International Archives of Medicine*, a novel scientific journal born in response to the new needs that have emerged as a result of globalization. The title of this journal reflects the profile and scope of the journal, as outlined below.

## Medicine

Current medical specialization makes it difficult to have a global view of medical practice, as investigators often restrict their research to the limits of their discipline but seldom beyond. In parallel, journals are becoming highly specialized and, with a few exceptions, are not aimed at a wide audience.

To optimize knowledge acquisition and usage, *International Archives of Medicine *aims to go beyond the limits of any given discipline and contribute to translational medicine, since the scientific process is meant, after all, to connect research with the reality of human biology and disease.

## Archives

Scientific and technological advances generate information at exponentially growing rates, making it necessary to organize that information in highly usable formats: integrated, graded and ranked. *International Archives of Medicine *aims to compile in its archives different types of articles focused on any medical speciality in three different formats. First is original research articles: it is important to regard research as a two-way process, from labs to bedside and from bedside to labs. Secondly, case reports: accurate, detailed recounting of clinical experience continues to be essential to the progress of medicine as a case report provides extensive and detailed information about an individual, which is often lost in larger studies. Case reports published in this journal will be aggregated into a database of medical case reports, a new initiative from BioMed Central to improve the usability of individual case reports. Finally, review articles: there is a great body of knowledge underpinning the conceptual and semantic barriers of subspecialties and systematic reviews are needed to integrate all this knowledge.

## International

The so-called "international" journals are rarely international in their content or readership. Most articles published in these journals focus on issues that are of importance primarily to Western audiences [1], and research from developing countries is under-represented in the main international medical journals [2]. In the current electronic era the possibilities for a medical journal to disseminate its content are almost limitless; it is not more difficult to provide access to readers from all around the world than to the readers from a single country [3]. *International Archives of Medicine *aims to reach a broad range of different countries throughout the five continents.

All content is freely open access [4], and therefore a country's economy will not influence its readers' ability to access articles. In addition, submission fees are waived for authors from countries with small economies (that are ranked in the World Bank's low income or lower-middle income categories) [5] to guarantee universal access for both readers and authors.

We have chosen a representative and diverse Editorial Board [6] including international experts in different fields of medicine to reflect the diversity of medicine today. This will help to bring the journal closer to authors and readers from different medical and geographically diverse areas.

An additional advantage for authors publishing in *International Archives of Medicine *is its relationship to *Archivos de Medicina *[7], which is the only Spanish language open access journal covering all areas of medicine. Many of the English language papers published in *International Archives of Medicine *will be translated and republished in *Archivos de Medicina *for the Spanish speaking audience, and vice versa.

*International Archives of Medicine *is a journal for the global medical community. We welcome contributions from the most sophisticated laboratories and from the most modest of medical-care settings. To fulfil this vision, manuscripts accepted after a peer review process will be immediately available online and promptly listed in PubMed to make this journal an important global resource. In line with our open access policy, authors retain the copyright of their work, allowing them to distribute their manuscript as they please [8]. We hope you will join us in this exciting venture and submit manuscripts to *International Archives of Medicine*.

## Competing interests

Manuel Menendez Gonzalez is the Editor-in Chief of *International Archives of Medicine*. BioMed Central has waived the article processing charge for this Editorial in line with the journal's contract.
